# Workplace narratives of South African employees with multiple sclerosis

**DOI:** 10.4102/ajod.v14i0.1725

**Published:** 2025-09-17

**Authors:** Armand Bam, Marco Bekker, Linda Ronnie

**Affiliations:** 1 Stellenbosch Business School, Stellenbosch University, Cape Town, South Africa; 2 School of Management Studies, University of Cape Town, Cape Town, South Africa

**Keywords:** multiple sclerosis, disability inclusion, sensemaking, social model, reflexivity, South Africa

## Abstract

**Background:**

Workplace inclusion for employees with chronic conditions such as multiple sclerosis (MS) remains a challenge because of the episodic and invisible nature of symptoms, leading to stigma, disclosure dilemmas and inadequate accommodations. Traditional approaches to disability inclusion often fail to address the lived realities of employees with MS, necessitating a deeper exploration of how individuals and organisations construct meaning around disability and inclusion.

**Objectives:**

This study explores how employees with MS experience workplace inclusion, self-management and disclosure drawing on sensemaking theory and the Social Model of Disability. The study also employs a reflexive approach, centring the voices of employees to generate actionable insights for employers and disability advocates.

**Method:**

An exploratory qualitative research design was adopted involving semi-structured interviews with 13 employees diagnosed with MS in South Africa. Thematic analysis was used to identify patterns related to workplace adaptability, disclosure and support structures. Researcher reflexivity was incorporated to acknowledge positionality and enhance the study’s depth.

**Results:**

Workplace adaptability, including flexible work arrangements and empathetic leadership, play a critical role in ensuring inclusion for individuals with MS in the work environment. However, disclosure remains a complex decision influenced by stigma and workplace culture. Psychological safety and proactive organisational sensemaking significantly impact employees’ experiences.

**Conclusion:**

For meaningful workplace inclusion, organisations must move beyond compliance-driven policies and foster an environment where employees with MS feel valued and supported.

**Contribution:**

By integrating reflexivity, sensemaking theory and the Social Model of Disability, this study offers valuable contributions to the discourse on disability inclusion.

## Introduction

Creating inclusive workplaces for individuals with chronic conditions such as multiple sclerosis (MS) has become a necessity for fostering equity, productivity and long-term employee well-being in the modern workplace (Vitturi et al. 2023). Multiple sclerosis is a complex autoimmune disease that is unpredictable in nature, characterised by the invisibility of its symptoms. Despite medical advancements, individuals with MS are faced with the stigma of being high-risk employees. Discrimination, inadequate accommodations and other barriers are exacerbated by the episodic nature of MS symptoms, which complicate conventional workplace structures and challenge traditional approaches to disability inclusion (Ståhl, Bjereld & Dunér 2022).

For most forms of disability, the decision of employees to disclose their disability to their employer presents dilemmas. In particular, employees with MS encounter barriers to their career progression and job security (Shahbaz & Parizad 2022), while employers’ approach to creating supportive environments call for more nuanced understandings of what constitutes meaningful inclusion (Kruger & Coetzee 2021). While research on workplace accommodations and disability policies has expanded in recent years (Bishop et al. 2025), studies focusing specifically on the lived experiences of employees with MS remain limited within the Global South and specifically in sub-Saharan Africa.

This study addresses these gaps by exploring how individuals with MS experience workplace inclusion, address issues of self-management and their propensity for disclosure. Drawing on sensemaking theory and the Social Model of Disability, this study explores the ways in which individuals with MS construct meaning around their work experiences and how managers and their respective environments either enable or curtail their participation.

A key element to this study is its reflexive approach, which acknowledges the position of the researchers while centring employees’ voices to arrive at actionable insights for employers and disability advocates. Through this qualitative inquiry, we highlight the need for managers to increase awareness of MS and normalise conversations about disability to ensure an inclusive workplace for employees with MS.

### Management of multiple sclerosis

Multiple sclerosis is a chronic neurological condition shaped by genetic, environmental and socioeconomic factors (Koch-Henriksen & Sørensen 2010). As of 2023, over 2.9 million people worldwide were living with MS, with South Africa reporting 4685 diagnosed cases (Atlas of MS n.d.). Symptoms range widely and include fatigue, visual disturbances, muscle weakness, sensory deficits and cognitive impairments (Induruwa, Constantinescu & Gran 2012). Clinical exacerbations, marked by sudden neurological deterioration, significantly influence disease progression and quality of life (QoL) (Schaeffer et al. 2015). While there is no cure for MS, disease management focuses on slowing progression, reducing relapse rates and alleviating symptoms. Supportive strategies such as physical rehabilitation, mental health interventions and lifestyle modifications – including exercise and dietary adjustments – are essential for enhancing functional abilities and overall QoL (Vosoughi & Freedman 2010).

Self-management is a vital strategy for enhancing the QoL for individuals living with MS. It emphasises personal responsibility and proactive strategies for controlling symptoms and improving overall well-being. A key element of self-management is self-efficacy – the confidence in one’s ability to perform health-related behaviours – which has been shown to significantly impact health outcomes and psychological well-being (Marks, Allegrante & Lorig 2005). Daily self-management practices, such as engaging in regular exercise, monitoring symptoms and maintaining communication with healthcare providers, empower individuals to manage their condition effectively.

Effective self-management is essential in the workplace, helping individuals sustain employment and productivity. Stress management techniques, including mindfulness and relaxation practices, reduce psychological distress while physical activity programmes and cognitive behavioural strategies provide structured support for managing work-related challenges (Muñoz San José et al. 2016; Reynard, Sullivan & Rae-Grant 2014; Strober & Arnett 2016). These multidisciplinary approaches integrate physical, psychological and social dimensions of care, empowering individuals with MS to lead healthier and more fulfilling lives (Simpson, Mair & Mercer 2015; Venasse, Edwards & Pilutti 2018).

### Sensemaking theory, the social model of disability and multiple sclerosis

Sensemaking theory (Weick 1995, 2005) and the Social Model of Disability (Oliver 2013, 2016) are complementary perspectives that can provide a framework for understanding how individuals with MS navigate workplace inclusion, self-management and disclosure. By illustrating how disability is socially constructed and how organisations can actively reshape their understanding of disability to foster inclusion, these theories shape the study’s analysis.

#### Sensemaking theory

Sensemaking theory (Weick 1995, 2005) explains how individuals and organisations construct meaning in ambiguous and challenging contexts. Applied to disability in the workplace, sensemaking provides insight into how individuals with MS interpret their experiences and how organisations react to disability disclosure, accommodation requests and employee support.

A key tenet of sensemaking is that people create order through retrospective meaning-making, where they continuously reconstruct narratives based on prior experiences and social interactions (Lee & McCormick 2002). For individuals with MS, this process involves weighing the risks and benefits of disclosure, interpreting organisational responses and navigating the evolving nature of their condition (Pakenham 2008). For organisations, sensemaking plays a role in shaping policies, attitudes and structures that either facilitate or hinder inclusion (Alvesson & Jonsson 2022).

In many workplace settings, disability is framed through a medical lens, wherein employees with chronic illnesses are perceived as requiring special accommodations rather than being valued as contributors to workplace diversity (Bunbury 2019). This narrow interpretation reinforces ableist narratives that equate disability with deficiency. Sensemaking theory suggests that organisations must actively reconstruct their understanding of disability, shifting from a reactive approach (merely accommodating employees with MS) to a proactive stance that normalises disability as part of workplace diversity.

#### The social model of disability

The Social Model of Disability (Oliver 2013, 2016) serves as a critical lens for examining how societal and environmental structures create disabling barriers for individuals with MS. Rather than positioning disability as an individual impairment, this model argues that exclusion results from structural, attitudinal and organisational barriers that limit participation (Barnes 2000).

In the context of workplace inclusion, the social model emphasises that accessibility and equity should not be contingent on disclosure but rather embedded into organisational culture (Bam 2025). By adopting this model, workplaces can move beyond a compliance-driven approach and focus on creating environments where individuals with MS can thrive without fear of discrimination.

Integrating the Social Model of Disability with sensemaking theory reveals the importance of organisational culture and leadership in shaping disability inclusion. If workplaces perceive MS as a legitimate aspect of workforce diversity, rather than a problem requiring accommodation, they create conditions where employees with MS feel valued and supported. This perspective encourages organisations to shift from viewing disability as an individual burden to recognising their own role in fostering inclusion.

#### Applying the conceptual framework to workplace inclusion, disclosure and self-management

The interplay between sensemaking theory and the Social Model of Disability is particularly relevant when analysing key workplace experiences of individuals with MS:

- **Workplace inclusion:** Organisations construct meaning around disability based on their cultural norms and policies. The extent to which they engage in inclusive sensemaking determines whether disability is normalised or stigmatised.
- **Disclosure:** The decision to disclose an MS diagnosis is shaped by how employees anticipate their workplace will interpret their condition. The risks of stigma and discrimination deter disclosure when organisations fail to create psychological safety.
- **Self-management:** Employees with MS develop self-management strategies to navigate fluctuating symptoms. However, these strategies are more effective when workplaces acknowledge invisible disabilities and proactively adapt workplace structures to accommodate diverse needs.

Using this conceptual framework, this study aims to explore the ways in which individuals with MS construct meaning around their work experiences in order to understand how employers and their respective environments either enable or curtail their participation.

## Research methods and design

This study adopted an exploratory qualitative research design to explore the lived experiences of individuals with MS in the workplace. The study’s exploratory nature aligns with its objective to uncover new insights into the complex challenges faced by individuals with MS, particularly in the context of disclosure, accommodations and inclusion. Grounded in a social constructivist paradigm, the study emphasises how participants construct meaning through their interactions and experiences, reflecting the socially constructed nature of disability. Constructivism, as articulated by Jafari Amineh and Davatgari Asl (2015), highlights that knowledge is created collaboratively, making it an appropriate framework for understanding how individuals with MS navigate their workplaces.

The qualitative approach enabled an in-depth exploration of subjective experiences, allowing for detailed accounts of participants’ perspectives. This design was particularly suited to understanding the nuanced and individualised ways in which MS impacts workplace dynamics and how participants manage the challenges that arise.

### Researcher reflexivity

Researcher reflexivity is essential in qualitative research, particularly when exploring sensitive topics (Ide & Beddoe 2024). Reflexivity requires researchers to critically examine their own biases, assumptions and positionality throughout the research process to ensure the authenticity and credibility of the findings (Jamieson, Govaart & Pownall 2023). In this study, one of the authors (A.B.) specialises in the research of the inclusion of people living with different forms of disability and applies a social justice lens to research. This perspective influences the study’s commitment to highlighting structural barriers, advocating for workplace equity and ensuring that participants’ voices are centred (Townsend & Cushion 2021). The second author (M.B.), is a person with MS and possesses a deeply personal understanding of the condition and its workplace challenges. The third author (L.R.) examines issues of diversity in the workplace from a management perspective and brings their experience, from both research and practice, to bear on the issue. This triad of positionality combined scholarly insights with lived experiences, which enriched the research process and required a conscious effort to balance advocacy with methodological rigour.

The second author conducted the interviews and was mindful of how their personal experience with MS could shape data collection and interpretation. To manage this, reflexive note-keeping was used throughout the research process. After each interview, the author recorded immediate thoughts, emotional reactions and any assumptions that surfaced. These reflexive notes helped distinguish the participant’s narrative from the author’s internal responses. They were not treated as data but were reviewed during analysis to flag potential bias and ensure that interpretation remained grounded in participants’ accounts.

The other authors were a critical sounding board and offered assurance in dedicated feedback sessions during data collection and analysis. In the debriefing sessions, the authors had the opportunity to articulate their personal realisations during the research process and to discuss any emotional or cognitive reactions that might influence interpretation. Observations were captured in writing, engagements were conducted through Socratic questioning to seek clarity, assumptions were challenged, evidence or reasoning for coding decisions were sought and alternative viewpoints of the experiences shared. Through these discussions, the authors were able to surface blind spots, challenge assumptions and strengthen the trustworthiness of the study and maintain reflexivity and transparency across all stages of the research. The sessions also offered assurance and confirmation of what author two observed in relation to the transcribed interviews.

Engaging with participants who faced severe workplace challenges and progressive symptoms of MS prompted deep reflection on the second author’s condition and future trajectory. While he had managed his symptoms relatively well, hearing firsthand accounts of worsening health, workplace discrimination and forced career adjustments led to an acute awareness that he, too, might 1 day face similar struggles. This realisation was both unsettling and illuminating, reinforcing his connection to the participants’ narratives while also heightening the need for continued advocacy and systemic change (Evans et al. 2017). At times, he found himself grappling with feelings of uncertainty and vulnerability, recognising that the challenges described in the study were not just theoretical but a possible reality for his own future (Sherry 2013). Despite this, he remained committed to ensuring that participants’ voices took precedence, using his positionality to foster trust while maintaining a critical distance in the analysis. This reflexive engagement strengthened the study’s depth, as the second author’s lived experience and newfound awareness contributed to a nuanced understanding of the urgency behind workplace inclusion efforts for individuals with MS.

Several strategies were employed to address positionality and enhance the study’s credibility. Recognising the potential power dynamics between interviewer and participant, the second author fostered open, trust-based conversations, drawing on shared experiences, where appropriate, to encourage deeper engagement. The authors themselves maintained individual reflexive journals to document their assumptions and emotional responses to participants’ narratives, ensuring that analysis was guided by the data rather than pre-existing beliefs (Jamieson et al. 2023). Member checking was used to validate key themes by sharing these with participants, allowing them to confirm or clarify interpretations. In addition, an audit trail was kept documenting all research stages, ensuring coding and thematic development transparency. The research team also engaged in bracketing, consciously setting aside personal biases to allow participants’ voices to take precedence.

### Participants

Participants were recruited through the MS Society of South Africa (Western Cape chapter) by leveraging their WhatsApp support groups to distribute invitations. The organisation provided consent for this approach, which ensured access to individuals with firsthand experience of MS in the workplace. A snowball sampling strategy was also employed, allowing participants to recommend others who met the inclusion criteria. This method provided an effective way to reach a diverse group while building trust through established networks.

To participate, individuals needed to be over 18 years of age, have been diagnosed with MS by a qualified physician for at least 3 years and be currently or previously employed in a full-time role. They were also required to be fluent in English, able to provide informed consent and have stable Internet access for virtual interviews. These criteria ensured the selection of participants with comparable levels of work-related experience and shared challenges. Individuals who were younger than 18 years of age, had only ever been self-employed or were unable to communicate fluently in English were excluded, as were those unable to provide written consent.

From the 26 persons who responded to the call, 13 individuals met the inclusion criteria and agreed to participate. This sample size provided a robust basis for exploring diverse yet comparable experiences while allowing for a detailed, in-depth analysis of each participant’s narrative. Demographic information was collected for all participants, and pseudonyms have been used to ensure confidentiality.

### Data collection

Data were collected through semi-structured interviews conducted virtually using video calls or telephone, depending on the participant’s preference and technological access. Semi-structured interviews offered the flexibility needed to explore participants’ unique experiences while maintaining a consistent framework across discussions. The interview guide was designed to cover key themes, including initial workplace inclusion, disclosure of MS diagnosis, accommodations and the broader challenges associated with navigating employment with MS.

### Data analysis

Thematic analysis, as outlined by Braun and Clarke (2006, 2023), was employed to analyse the data from the transcribed interviews systematically. Iterative, inductive coding was used to allow the themes to emerge directly from the narratives of participants, rather than pre-imposed categories. While coding was inductive, both sensemaking theory and the Social Model of Disability served as guiding theoretical lenses in interpreting the data rather than as *a priori* coding frameworks. These theories informed the analytical focus by helping the researchers understand how participants made meaning of their workplace experiences (sensemaking) and how structural barriers shaped these experiences (social model). The theories were used post-coding to interpret and frame the emergent themes rather than to direct the initial coding process.

The first step involved familiarisation with the data, where transcripts were read and re-read to immerse the authors in the participants’ narratives. This process allowed initial patterns and recurring ideas to emerge naturally. In the next phase, the data were systematically coded by identifying meaningful segments of text related to the study’s objectives, such as ‘barriers to inclusion’ or ‘impact of disclosure’.

Codes were then systematically grouped into broader themes that captured the central patterns across participants’ experiences. For instance, codes related to remote work, flexible schedules and task prioritisation were categorised under ‘Workplace adaptability and disclosure’, reflecting the importance of workplace flexibility in managing MS symptoms. Similarly, experiences of stigma, workplace culture and disclosure challenges were integrated into this theme.

Each theme was reviewed to ensure coherence and alignment with the raw data, refining overlapping or redundant themes for clarity. The themes were then clearly defined and named to accurately represent their underlying concepts, such as ‘Support systems and MS challenges’, which encompassed the role of empathetic leadership, psychological safety, resilience, fatigue and workplace adaptations in fostering an inclusive work environment.

Finally, these themes were integrated into a cohesive narrative that highlights both the shared and unique experiences of participants. Direct quotes were included to ensure that participants’ voices were authentically represented in the final analysis, reinforcing the nuanced challenges and strategies they employed in navigating the workplace with MS.

### Trustworthiness

To ensure the rigour of our qualitative research, Lincoln and Guba’s (1985) model of trustworthiness was used to give attention to the dimensions of credibility, dependability, transferability and confirmability. Credibility was enhanced through prolonged engagement with the data and participants. Interviews were conducted over an extended period, and follow-up interactions allowed participants to clarify or expand on their accounts. Member checking was used to verify interpretations by sharing transcripts and preliminary findings with participants to ensure accurate representation of their narratives.

To ensure dependability, a detailed audit trail was created that documented decisions throughout the research process, from interview protocols to coding steps and theme development. Researcher triangulation was also used to strengthen dependability, limit individual bias and ensure a more comprehensive understanding of the participants’ experiences. All authors independently coded the transcripts, compared codes, discussed discrepancies and refined the coding frame through consensus.

To support transferability, thick descriptions were used to provide contextual detail about each participant’s background, employment setting and disability experience. This allows readers to assess the applicability of findings to other contexts.

To ensure confirmability, all analytic decisions and reflexive insights were recorded in a research journal. Data interpretations were grounded in direct quotations from participants, and author triangulation reduced the influence of individual bias on theme development.

### Ethical considerations

Ethical approval to conduct this study was obtained from the Stellebosch University, Social, Behavioural and Education Research ethics committee (Project no: 31339). All procedures followed established ethical guidelines as stipulated by the committee. Written informed consent was obtained, and participants were fully informed about the study’s purpose, voluntary nature and their right to withdraw at any stage without consequence. Confidentiality was strictly maintained, with data anonymised and pseudonyms assigned to protect participant identities. No financial compensation was provided, and all interviews were conducted in a private setting to ensure participant comfort and openness. Transcriptions were securely stored on the university’s password-protected OneDrive to ensure data confidentiality and integrity. Given the varied nature of MS, ethical considerations prioritised individual autonomy over general assumptions about vulnerability. All participants were or had been employed and were capable of informed decision-making. Ethical oversight focused on their abilities rather than broad disability-related perceptions.

## Results

The study participants included 13 people of varying demographics (in terms of age and gender) and experiences with MS (in terms of MS type, years since diagnosis and disclosure status) (Table 1). The findings from the semi-structured interviews coalesced around two themes: workplace adaptability and disclosure, and support systems and MS challenges. Multiple sub-themes emerged (Table 2), which will be explored in detail.

**TABLE 1:** Participant demographics.

Participant	Age (years)	Gender	Province	Country of birth	Type of MS	Disclosure status	Years since diagnosis	Employment status at time of study
Emily	48	Female	Gauteng	South Africa	Primary Progressive	Partial or None	15	Employed Full-time
James	32	Male	Western Cape	South Africa	Relapsing-Remitting	Full	4	Employed Full-time
Amy	47	Female	Western Cape	South Africa	Relapsing-Remitting	Full	32	Employed Full-time
Jess	29	Female	Western Cape	South Africa	Relapsing-Remitting	Full	3	Self-Employed
Jane	39	Female	Western Cape	South Africa	Relapsing-Remitting	Full	5	Employed Full-time
Chris	62	Male	Western Cape	South Africa	Relapsing-Remitting	Full	3	Employed Full-time
Jackie	36	Female	Western Cape	South Africa	Relapsing-Remitting	Partial	2	Employed Full-time
Paul	68	Male	Gauteng	Switzerland	Relapsing-Remitting	Full	3	Self-Employed
Karen	41	Female	Western Cape	South Africa	Relapsing-Remitting	Full	13	Unemployed
Adam	47	Male	Western Cape	South Africa	Relapsing-Remitting	Full	29	Employed Full-time
Melissa	51	Female	Western Cape	South Africa	Relapsing-Remitting	Full	22	Self-Employed
John	31	Male	Western Cape	South Africa	Relapsing-Remitting	Full	5	Employed Full-time
Lauren	62	Female	Gauteng	Portugal	Relapsing-Remitting	Full	11	Employed Full-time

MS, multiple sclerosis.

**TABLE 2:** Summary of interview themes and key findings.

Theme	Subtheme	Key findings	Sample quotes
Workplace adaptability and disclosure	Remote work and flexible hours	Remote work and flexibility were essential for managing MS symptoms, reducing stress and improving productivity.	‘Working from home is a blessing … I get to hide the physical side of it.’ (Emily, 48, female, Gauteng)
	Task prioritisation	Task prioritisation allowed participants to align work with peak energy levels, yet rigid expectations made this difficult.	‘When I take my Nuvigil in the morning, that’s like my golden period.’ (Jess, 29, female, Western Cape)
	Isolation challenges	While remote work was beneficial, it sometimes resulted in professional and social isolation, impacting engagement and support.	‘Working in complete isolation is … not suitable.’ (Jess, 29, female, Western Cape)
	Stigma and workplace culture	Workplace stigma often deterred disclosure, making it harder to access accommodations without fear of judgement.	‘I got labelled; I was the one that shouldn’t be touched.’ (Emily, 48, female, Gauteng)
	Disclosure risks and benefits	Disclosure was a double-edged sword; while some participants benefited from employer understanding, others faced discrimination.	‘Disclosure builds trust when it’s met with support.’ (John, 31, male, Western Cape)
Support systems and MS challenges	Empathetic leadership	Supportive leadership created an inclusive environment where employees with MS felt valued and supported.	‘The entire office culture trickled down from my manager [*who was*] always willing to listen.’ (Karen, 41, female, Western Cape)
	Psychological safety	Psychological safety was key in fostering trust and allowing employees to discuss their challenges without fear of reprisal.	‘The company needs to create that psychologically safe space so I can say, “this is the situation”.’ (Chris, 62, male, Western Cape)
	Fatigue and cognitive impairments	Fatigue and cognitive impairments significantly affected workplace performance, requiring better employer awareness and support.	‘It’s almost like a fatigue that you can’t express … it’s a severe fatigue that impacts everything.’ (Jess, 29, female, Western Cape)
	Mental health and resilience	Mental health was a critical factor in workplace engagement, as stress and anxiety exacerbated MS symptoms.	‘Mental health isn’t optional – it’s the key to everything.’ (Paul, 68, male, Gauteng)
	Workplace adaptations	Workplace adaptations, including ergonomic tools and cognitive aids, were underutilised but crucial for productivity and retention.	‘Cognitive challenges don’t mean incompetence; they mean adaptation.’ (Amy, 47, female, Western Cape)

MS, multiple sclerosis.

### Workplace adaptability and the challenge of disclosure

The necessity for workplace adaptability emerged as a fundamental theme, encompassing both the structural flexibility required to accommodate MS symptoms and the challenges associated with disclosing a diagnosis in the workplace. Traditional workplace structures often failed to account for the fluctuating nature of MS, where rigid schedules exacerbated symptoms such as fatigue and cognitive challenges. The findings indicate a shared recognition among participants that workplace flexibility – encompassing remote work, adaptable hours and the ability to prioritise tasks based on individual capacity – was not merely a preference but a necessary accommodation for managing their condition effectively. Without such flexibility, productivity declined and individuals faced additional stress in managing their symptoms. Jess (29, female, Western Cape) specifically raised the misalignment between the hours of her peak productivity and her employers’ expectations regarding her working hours:

‘When I take my Nuvigil in the morning, that’s like my golden period until one o’clock … they prefer to schedule meetings with clients after hours. That’s not viable for me, by six o’clock, I can’t do it anymore.’

Remote work was frequently cited as a key enabler of productivity and wellbeing. Lauren (62, female, Gauteng) could ‘operate at an optimal level when working from home’. The ability to have control over her work productivity rhythm was highlighted during the coronavirus disease 2019 (COVID-19) pandemic when she was able to ‘get up at five o’clock in the morning’ and complete a day’s work before her actual office hours began. What she felt was key to the autonomy to make the decision to ‘stop for half an hour’ without feeling judged. In this way Lauren and other participants could structure the day around their health needs. In turn, participants like Emily (48, female, Gauteng) felt that work-from-home options were a ‘blessing’ as she could ‘hide the physical side of it’. This was not the case when having to ‘go to the office … and find a way of walking without assistance’. She captured all the participants’ common experience of being ‘exhausted’ by having to conceal and ‘hide it’.

Workplace adaptability also came with challenges. Participants who worked in isolation experienced a lack of support that also compounded their difficulties. Jess (29, female, Western Cape) explained ‘working in complete isolation is … not suitable’ and posed a significant risk for the business if she had a ‘relapse and was admitted to the hospital [*because*] there was no support’, yet sick leave was ‘frowned upon’.

The issue of disclosure was also closely linked to workplace adaptability. While disclosure could lead to accommodations and understanding, the risks of stigma and discrimination increased. Emily (48, female, Gauteng) hesitated to disclose her MS diagnosis because of concerns about being perceived as less capable or facing negative career consequences. She was ‘labelled the one that shouldn’t be touched … was stupid, and fat people get diseases like that because they don’t look after themselves’. This degradation led to the conscious decision to conceal further disclosure at her current employer ‘to try and avoid the hurt that comes with being labelled’.

The visibility of Jackie’s (36, female, Western Cape) symptoms compelled her disclosure as she wanted to avoid her ‘employer thinking something else is going on’. James (32, male, Western Cape) suggested it was ideal to have all his ‘ducks in a row and the best medical help’ and to keep his employer informed of what medication and treatment he was on giving them constant feedback on ‘how this is going to impact the organisation and myself’. The effectiveness of adaptable working conditions relied heavily on an environment that supported disclosure without fear of stigma. Adam (47, male, Western Cape) considered that ‘disclosure built trust when it was met with support’. In other words, participants wanted organisations to create a culture supporting safety and accommodations in order to maintain their job security and professional reputations.

### Support systems and multiple sclerosis challenges

The role of support systems, both within and outside the workplace, was another central theme. Participants emphasised that workplace culture, leadership attitudes and access to understanding colleagues impacted their ability to navigate employment successfully. Empathetic leadership and open communication were identified as key enablers of inclusion, with managers playing a crucial role in shaping workplace attitudes towards MS. In Karen’s case, the ‘entire office culture trickled down by that good manager … always willing to listen and willing to help and say, “Okay, what can I do?”’ James (32, male, Western Cape) considered that in smaller organisations with limited support structures managers, needed ‘social and emotional intelligence’ calling for an ‘adaptive leadership approach’ and the understanding of what staff with MS would need. Similarly, Karen (41, female, Western Cape) supported the motion that it was emotional intelligence of leaders ‘that makes the difference between surviving and thriving’. The inclination to disclose was increased where participants felt ‘psychological safety’. Chris (62, male, Western Cape) noted that he would feel a sense of being supported if his employers were open to ‘work through this and see what the best for me is as an individual’. Community-based support groups were also essential in enhancing the employment experience, but Chris wanted them to be ‘positive instead of showing the negative sides of MS’.

Fatigue and cognitive challenges were among the most difficult aspects of MS for participants to manage, significantly impacting their work performance and social interactions. Unlike visible symptoms, these impairments were often misunderstood, leading to additional workplace challenges. Jess (29, female, Western Cape) described it as more than just tiredness but ‘severe fatigue that impacts [*your*] ability to talk and think’. She also discussed her difficulty with memory and focus because of being ‘severely numb cognitively’. Lauren (62, female, Gauteng) also highlighted the mental health dimension of managing her MS, claiming mental health was not optional because ‘it’s the key to everything’.

Participants like Jackie (36, female, Western Cape) developed their own coping mechanisms and because of her ‘cognitive issues’ she had to be alert and ‘immediately write down or type out’ what was transpiring or required in meetings otherwise she would forget the detail. While fatigue constrained participants physically, Jackie was adamant that ‘cognitive challenges didn’t mean incompetence, it meant adaptation’. Calling on better workplace support, Paul (68, male, Gauteng) was mindful that just because MS was invisible, it did not mean it was ‘non-existent’ and that his struggles were real and tangible, noting that ‘education on MS should be part of every company’s onboarding process’. Participants showed remarkable resilience in overcoming systemic barriers; however, they grappled with feelings of self-doubt when faced with workplace limitations. Jess (29, female, Western Cape) explained her disappointment as she had ‘studied for 8 years, earned a master’s degree, and now I’m doing admin work. It’s hard not to feel like I’ve lost part of myself’. While this was a shared sentiment for other participants. James (32, male, Western Cape) highlighted the power or purpose at the point when he was at his lowest: ‘[*my*] daughter was my reason to get up every morning. Multiple sclerosis isn’t a death sentence; there’s still a life to live after the diagnosis’.

## Discussion

This study provides new insights into the workplace experiences of individuals living with MS, offering a nuanced understanding of how structural barriers, self-management strategies and organisational culture intersect to shape their professional trajectories. The findings contribute to existing literature on disability and employment by centring the voices of individuals with MS and incorporating a reflexive research approach that enhances the depth and reliability of the analysis. By integrating sensemaking theory and the Social Model of Disability, the study broadens conventional discussions on workplace accommodations, demonstrating how organisations construct and reconstruct their understanding of disability and inclusion.

### Sensemaking, workplace adaptability and disclosure

The study contributes to the literature by integrating sensemaking theory (Weick 1995, 2005) and the Social Model of Disability (Oliver 2013, 2016) to explain how individuals with MS and their employers construct meaning around disability, disclosure and accommodations. The decision to disclose emerged as one of the most complex challenges participants face, as it requires navigating personal, organisational and societal interpretations of MS. Consistent with previous research (Reed et al. 2017), findings show that individuals weigh the potential benefits of disclosure – such as receiving accommodations and fostering trust – against the risks of stigma and discrimination.

From a sensemaking perspective, workplace disclosure is not merely an individual decision but a socially constructed process, shaped by organisational culture and leadership attitudes. Participants described comfort with disclosure when clear organisational support structures were in place, reinforcing the idea that disclosure outcomes depend on how well organisations engage in inclusive sensemaking. This echoes Clair, Beatty and Maclean (2005) argument that disclosure should not be framed as a ‘risk’ borne by employees alone, but as a process that organisations must actively facilitate through psychological safety and transparent accommodation policies.

Workplace adaptability also emerged as a critical but underutilised factor in promoting employment retention for individuals with MS. Participants emphasised the necessity of flexible work arrangements, aligning with Muñoz San José et al.’s (2016) finding that autonomy in work schedules significantly improves employment retention for individuals with chronic conditions. However, as some participants explained, flexibility alone is insufficient if organisations do not actively challenge ableist narratives that equate legitimacy with visibility when it comes to disability.

By framing these workplace challenges within sensemaking theory, this study highlights that organisations must not only implement accommodations but also reshape their collective understanding of disability. This also corresponds to the Social Model of Disability, emphasising that barriers exist not because of individual impairments but because of workplace cultures that fail to adapt to diverse needs (Oliver 2013, 2016).

### Fatigue, cognitive challenges and psychological safety

This study reinforces the importance of psychological safety in shaping the workplace experiences of individuals with MS. Participants repeatedly emphasised that supportive leadership, open communication and emotionally intelligent managers significantly impacted their ability to navigate employment because they set culture. This finding concurs with Edmondson’s (2018) research on psychological safety, which demonstrates that employees are more likely to disclose challenges and seek support in workplaces where trust is embedded in organisational culture.

The impact of fatigue and cognitive challenges also emerged as a major workplace barrier, reinforcing previous findings (Reynard et al. 2014) that MS-related fatigue is often misunderstood or dismissed by employers. Participants described how their fatigue affects all aspects of their lives, underscoring how neurological impairments can be invisible in workplace settings. The findings suggest that existing workplace accommodations tend to focus on physical accessibility rather than cognitive and energy-related accommodations, leaving individuals to self-manage their challenges without institutional support.

From a sensemaking perspective, these findings highlight the need for organisations to engage in a more nuanced understanding of disability. Rather than perceiving MS-related fatigue as an individual performance issue, workplaces should reframe their approach to inclusion by integrating education on invisible disabilities into managerial training and onboarding processes (Venasse et al. 2018). This aligns with the broader shift in disability inclusion literature, which calls for policies that anticipate rather than merely react to employee needs (Thompson et al. 2022).

### Expanding the literature through reflexivity and positionality

Researcher reflexivity played a crucial role in strengthening the credibility and depth of this study, particularly in acknowledging the positionality of the research team. The first author’s expertise in disability inclusion and social justice shaped the study’s emphasis on structural and policy-level barriers, ensuring that the research did not individualise workplace challenges but rather situated them within systemic inequities. The second author’s lived experience with MS brought an additional layer of depth, facilitating an environment of mutual trust during interviews and allowing for deeper engagement with participants. The third author’s expertise and experience with organisational behaviour and people management ensured that diversity and workplace inclusion were approached and analysed from a managerial perspective.

This approach concurs with Finlay’s (2002) perspective on reflexivity as a means of enhancing research transparency and acknowledging the researcher’s influence on knowledge production. By maintaining reflexive journals, engaging in ongoing discussions about researcher bias and employing bracketing techniques, the study mitigated the risk of preconceived notions shaping the analysis. This methodological rigour contributes to the broader disability research landscape by demonstrating how lived experience and scholarly expertise can coexist in research, leading to richer, more authentic insights.

In addition, the second author’s realisation that he might eventually face the same workplace struggles as the participants introduced an autoethnographic element to the study, reinforcing the personal and evolving nature of disability experiences. This echoes the work of Ellis, Adams and Bochner (2011), who argue that personal narratives in research can serve as powerful tools for understanding complex social phenomena, particularly in disability studies. By integrating both scholarly distance and personal connection, this study offers a model for future research that values both empirical rigour and researcher introspection.

### Contribution and limitations

By integrating reflexivity, sensemaking theory and the Social Model of Disability, this study offers valuable contributions to the discourse on disability inclusion. Using researcher reflexivity and positionality, this study demonstrates how lived experience and scholarly analysis can come together without compromising methodological rigour. The deliberate engagement with reflexivity ensures that the complexities of researching chronic illness in the workplace are acknowledged, allowing for a more nuanced and authentic representation of participants’ experiences. By recognising the interplay between personal bias and academic objectivity, the study strengthens the qualitative research process, offering a model for future inquiries into disability and employment.

The application of sensemaking theory provides a novel framework for understanding disclosure dilemmas, organisational culture and workplace inclusion. Rather than viewing disclosure as an individual act, the Social Model of Disability emphasises the collective process through which employees and employers construct meaning around disability. By examining how workplace policies and leadership responses influence the decision to disclose, this study extends previous research on disability in employment, offering insights into the social and organisational factors that shape these experiences. A critical takeaway is the need for organisations to move beyond surface-level accommodations and engage in meaningful dialogue that reshapes workplace narratives around disability.

Findings suggest that workplace policies must transcend compliance-driven approaches and actively foster inclusive sensemaking. Managers and leaders must, themselves, have awareness of the full range of disabilities their employees may experience, especially invisible disabilities, and the challenges associated with them. Managers need to normalise conversations about disability and chronic illness within organisations to create environments where disclosure is met with understanding rather than scepticism. Anticipatory accommodations, rather than reactive measures, can support employees in managing their health while maintaining their professional contributions. By shifting organisational narratives and embedding disability inclusion into workplace culture, companies can create a more supportive and sustainable work environment for individuals with MS and other chronic conditions.

Future research should explore how inclusive sensemaking evolves over time within organisations and assess whether managerial training interventions can shift workplace attitudes towards chronic illness. Longitudinal studies tracking the career trajectories of employees with MS would provide further insight into how workplace accommodations impact long-term employment outcomes. As this study only included participants from two provinces, the findings may not be transferable to other regions. Future research should expand the participant pool to include employees with MS from across South Africa. As organisations strive for greater inclusivity, ongoing research will be essential in ensuring that disability inclusion moves beyond policy rhetoric to become an embedded and lived reality within the workplace.

## Conclusion

This study provides a reflexive, theory-driven and empirically grounded exploration of the workplace experiences of individuals with MS. By integrating the Social Model of Disability, sensemaking theory and researcher reflexivity, it offers a multidimensional perspective on disclosure, accommodations and inclusion. As the Social Model of Disability stipulates, organisations play a pivotal role in constructing and reconstructing understandings of disability. The findings of this study emphasise that inclusive workplaces require more than just structural adjustments if they demand a shift in how disability is understood and valued within organisations. Leaders, managers and employees collectively shape workplace culture through policies, attitudes and interpersonal interactions.

Inclusive sensemaking requires organisations to actively challenge ableist assumptions and create environments that prioritise dialogue, flexibility and structural accessibility. This involves training managers and employees to recognise the fluctuating and invisible nature of MS, moving beyond legal compliance to foster proactive inclusion, and creating clear, supportive policies for disclosure that reduce stigma. In addition, it requires encouraging the co-creation of policies with employees with MS to ensure their lived experiences inform workplace structures. Through active sensemaking, leadership engagement and proactive policy development, workplaces can move beyond accommodation towards genuine inclusion, ensuring that individuals with MS feel supported, valued and empowered to thrive within organisations.
